# Plasma Proteomic Kinetics in Response to Acute Exercise

**DOI:** 10.1016/j.mcpro.2023.100601

**Published:** 2023-06-19

**Authors:** Michael Y. Mi, Jacob L. Barber, Prashant Rao, Laurie A. Farrell, Mark A. Sarzynski, Claude Bouchard, Jeremy M. Robbins, Robert E. Gerszten

**Affiliations:** 1 Division of Cardiovascular Medicine, Beth Israel Deaconess Medical Center, Boston, Massachusetts, USA; 2 CardioVascular Institute, Beth Israel Deaconess Medical Center, Boston, Massachusetts, USA; 3 Department of Exercise Science, Arnold School of Public Health, University of South Carolina, Columbia, South Carolina, USA; 4 Human Genomics Laboratory, Pennington Biomedical Research Center, Baton Rouge, Louisiana, USA

**Keywords:** acute exercise, exercise treadmill test, proteomics, plasma, circulation, HERITAGE Family Study

## Abstract

Regular exercise has many favorable effects on human health, which may be mediated in part by the release of circulating bioactive factors during each bout of exercise. Limited data exist regarding the kinetic responses of plasma proteins during and after acute exercise. Proteomic profiling of 4163 proteins was performed using a large-scale, affinity-based platform in 75 middle-aged adults who were referred for treadmill exercise stress testing. Plasma proteins were quantified at baseline, peak exercise, and 1-h postexercise, and those with significant changes at both exercise timepoints were further examined for their associations with cardiometabolic traits and change with aerobic exercise training in the Health, Risk Factors, Exercise Training and Genetics Family Study, a 20-week exercise intervention study. A total of 765 proteins changed (false discovery rate < 0.05) at peak exercise compared to baseline, and 128 proteins changed (false discovery rate < 0.05) at 1-h postexercise. The 56 proteins that changed at both timepoints included midkine, brain-derived neurotrophic factor, metalloproteinase inhibitor 4, and coiled-coil domain-containing protein 126 and were enriched for secreted proteins. The majority had concordant direction of change at both timepoints. Across all proteins assayed, gene set enrichment analysis showed increased abundance of coagulation-related proteins at 1-h postexercise. Forty-five proteins were associated with at least one measure of adiposity, lipids, glucose homeostasis, or cardiorespiratory fitness in Health, Risk Factors, Exercise Training and Genetics Family Study, and 20 proteins changed with aerobic exercise training. We identified hundreds of novel proteins that change during acute exercise, most of which resolved by 1 h into recovery. Proteins with sustained changes during exercise and recovery may be of particular interest as circulating biomarkers and pathways for further investigation in cardiometabolic diseases. These data will contribute to a biochemical roadmap of acute exercise that will be publicly available for the entire scientific community.

## Highlights

• Profiled 4163 plasma proteins at rest, peak exercise, and 1-h after exercise.
• Identified large proteomic perturbations at peak exercise that resolve 1-h later.
• Observed increased coagulation-related proteins post exercise but not at peak.
• Proteins with sustained changes were related to cardiometabolic traits.

## Graphical Abstract

**Figure:**
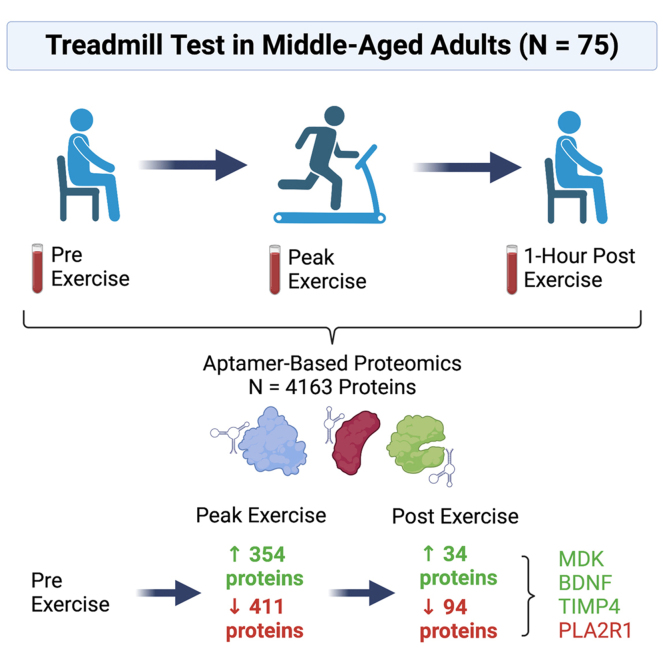

## In Brief

After measuring 4163 circulating proteins before, during, and 1-h after treadmill exercise in 75 middle-aged adults, we found that 765 proteins changed at peak exercise, and the majority of these proteins returned to baseline abundance 1-h after. Of 56 proteins with sustained change at peak exercise and after rest, there was enrichment for known secreted proteins and association with cardiometabolic traits in an independent cohort, suggesting that these proteins may be promising candidates for further investigation.

Regular exercise provides numerous health benefits including favorable cardiometabolic adaptations (1, 2), reduced risk of chronic diseases (3, 4), and lower all-cause mortality (5). Even a single bout of exercise can improve glucose uptake (6), lipid oxidation (7), and blood pressure (8), which may translate into sustained improvements in health over repeated exposures. However, despite decades of research enumerating exercise’s health benefits, the molecular mechanisms responsible for the adaptations—both acute and chronic—remain incompletely understood (9). Various studies have shown that acute exercise stimulates the release of bioactive factors into circulation, such as lactate (10), interleukin-6 (11), irisin (12), β-aminoisobutyric acid (13), and N-lactoyl-phenylalanine (14) that may mediate exercise’s biological effects. These “exerkines” are the source of intense investigation as the scientific community begins to unravel the biochemical pathways that are influenced by exercise (15, 16).

Plasma proteins represent a promising resource for studying exercise as their abundance reflects the systemic physiological state of an individual (17), and circulating proteins have been consistently identified as being responsive to exercise (15, 18). Prior studies have demonstrated broad protein translation and degradation responses to acute exercise (19, 20), and recent works applying plasma proteomics during acute exercise have identified distinct proteins or proteoforms altered in response to a single exercise bout (21, 22, 23). However, these investigations have largely focused on either a single, intra-exercise timepoint (21) or a relatively small number of proteins (22, 23) and thus have provided less insights into postexercise plasma protein kinetics and broader proteomic changes. Additionally, while adaptations to regular exercise arise through successive bouts of exercise (19), it remains uncertain whether exercise’s acute effects on plasma proteins would translate to chronic training-induced proteomic changes.

Therefore, the goal of the current study was to investigate large-scale plasma proteomic changes that occur during acute exercise as well as in the postexercise period. We present data on the changes of 4163 human proteins assessed by an affinity-based proteomics platform in a real-world, hospital-based cohort that was referred for treadmill exercise stress testing. We examined whether protein changes that occur at peak exercise remain sustained an hour into recovery and whether proteins with sustained changes relate to cardiometabolic traits and change with chronic exercise training in an independent cohort without overt cardiometabolic disease.

## Experimental Procedures

### Acute Exercise Cohort

We recruited 75 patients who were referred for cardiac stress testing at the Massachusetts General Hospital between January 2006 through December 2014. All participants underwent a symptom-limited treadmill exercise stress test according to a standard Bruce protocol (3-min stages with increase in treadmill speed and incline at each stage) (24) and had nuclear perfusion imaging. We excluded any individual who had evidence of myocardial ischemia based on a composite assessment of symptoms, electrocardiography, and nuclear myocardial perfusion imaging. We obtained demographic and clinical information at the time of stress test by patient’s self-report and review of medical record. Cardiovascular disease (CVD) was defined by a history of myocardial infarction, coronary revascularization, congestive heart failure, stroke, or peripheral arterial disease. Standard laboratory data were measured as part of routine clinical care and obtained from medical records. We collected plasma samples immediately prior to exercise, at peak exercise when the test was stopped, and at 1 h after the end of exercise through peripheral intravenous catheters. Samples were collected in standard K2 EDTA-treated 10 ml vacutainer tubes, immediately centrifuged at 3100 rpm for 10 min, and stored in −80 °C. The study was approved by the Institutional Review Boards of the Massachusetts General Hospital and Beth Israel Deaconess Medical Center and abided by the Declaration of Helsinki principles. All participants provided written informed consent.

### HERITAGE Family Study

The HEalth, RIsk factors, exercise Training And GEnetics (HERITAGE) Family Study was a 20-week, single-arm, aerobic exercise interventional study conducted between 1992 and 1997 (25). It enrolled 855 sedentary (*i.e.*, no regular physical activity for at least 3 months) participants between 17 and 65 years of age from 218 two-generation nuclear families of self-identified White and Black individuals across four North American sites. Participants were excluded if they had any significant medical conditions and diseases. No participants took medications to lower blood pressure, lipids, or glucose. Participants underwent detailed cardiopulmonary and cardiometabolic phenotyping before and after exercise training, which included the following: body composition measurements by computed tomography and underwater weighing, maximal effort cardiopulmonary exercise test, and frequently sampled intravenous glucose tolerance test (26). Participants exercised on average three sessions per week at an intensity and duration that was gradually increased over 20 weeks based on their maximum oxygen uptake (VO_2_max) measured at the baseline cardiopulmonary exercise test. Full details of the research protocol and phenotypic measurements are summarized in a review of HERITAGE (25).

### Proteomic Profiling

Detailed methods of the aptamer-based SomaScan (SomaLogic, Inc) are previously described (27, 28, 29). In brief, the assay binds target proteins with modified ssDNA aptamers that have slow on-off kinetics. The aptamer–protein complexes are then captured and recaptured by streptavidin beads in a two-step process. The number of bound aptamers is measured on a microchip array by activation of fluorophore tags attached to the beads and quantified as relative fluorescence units (RFUs), which are proportional to protein abundance. RFUs are normalized by plate and to reference calibrators in a multistep process to remove variation due to hybridization and sample manipulation across plates and runs. Previous work by our group showed that the mean intra-assay coefficient of variation and inter-assay coefficient of variation for the platform are approximately 7.5% and 6.5%, respectively (30). Prior to analysis, we log_2_-transformed normalized RFUs and then standardized values to mean 0 and SD 1. We profiled samples from the acute exercise cohort in two batches, approximately 10 months apart, and standardized RFUs separately within each batch to adjust for interbatch variations. All three timepoints for a single participant were assayed within the same batch. Acute exercise plasma samples had zero prior freeze-thaw cycles, and HERITAGE samples had 0 or 1 prior freeze-thaw cycle. supplemental Fig. S1 shows the distribution of correlations between storage duration in days and aptamer fold change. There were no missing aptamer values in the acute exercise cohort. Missing aptamer values in HERITAGE were not imputed.

### Aptamer-Protein–Binding Specificity

We collated supportive evidence of each aptamer’s specificity of binding to its target proteins from several sources. First, we use published genome-wide association studies of the SomaScan platform to describe whether an aptamer has *cis* protein quantitative trait loci (pQTLs), that is, genetic variants located within 1 Mb of the cognate protein’s transcription start site that correlate with its aptamer probe’s abundance (31, 32, 33, 34, 35, 36, 37, 38). We additionally performed proteomics profiling and assayed 1463 unique proteins using an antibody-based method (Olink) in 209 baseline samples from HERITAGE (28, 29). In brief, Olink’s technology utilizes two distinct polyclonal antibodies that have oligonucleotide sequence tags to bind a target protein, and the oligonucleotide sequences hybridize when brought into proximity, which are amplified and analyzed by next-generation sequencing to determine protein abundance (39). A detailed comparison of the SomaScan and Olink Explore platforms and their methods in HERITAGE are previously described (29). In addition to our findings in HERITAGE, we reported Spearman correlations (40, 41) and Pearson correlations (42) from three other studies that systematically compared the two platforms as well.

### Gene Set Enrichment Analysis

Gene set enrichment analysis (GSEA) of proteins altered either during peak exercise or 1-h postexercise was performed using the R package *fgsea* version 1.24.0 (https://bioconductor.org/packages/release/bioc/html/fgsea.html). Beta coefficients for fold change were ranked in descending order as inputs, and each timepoint was analyzed separately. Hallmark (43) and gene ontology (GO) gene sets were downloaded from MsigDB (https://www.gsea-msigdb.org/gsea/msigdb/index.jsp) in March 2023. To account for selected proteomic coverage by the SomaScan platform, we used a custom background for each gene set to include only genes corresponding to proteins measured on the platform. Empiric *p*-values were calculated based on 100,000 permutations, and significant enrichment was defined by the Benjamini–Hochberg false discovery rate (FDR) <0.05 across the number of gene sets tested.

### Experimental Design and Statistical Rationale

Plasma was collected from 75 participants at baseline, peak exercise, and 1-h postexercise for a total of 225 samples. The SomaScan version utilized in this study included 4789 aptamers that targeted 4163 distinct proteins with 546 proteins having more than one aptamer probe. For proteins with more than one aptamer probe, we selected the aptamer with the most statistically significant change (*i.e.*, smallest *p*-value) at a specific timepoint to represent that protein under the assumption that a poorer performing aptamer would capture more noise and less true changes. We compared protein abundance at peak exercise and 1-h after exercise to baseline abundance using a two-sided, one-sample *t* test. To determine significance, we corrected for multiple hypothesis testing for each timepoint separately using FDR <0.05 across 4789 aptamers tested. Proteins that changed significantly at either exercise timepoint were related to baseline clinical characteristics using age- and sex-adjusted linear regression models. In exploratory analyses to account for the varying degrees of exercise work performed, we further tested whether these proteins’ changes at peak exercise or 1-h postexercise had a relationship with metabolic equivalent of task (MET) achieved using a linear model that adjusted for baseline protein abundance, age, sex, and body mass index (BMI).

We subsequently extended our acute exercise findings in HERITAGE, an endurance exercise training study that tested the effects of chronic exercise. At baseline, 764 sedentary but otherwise healthy HERITAGE subjects underwent baseline proteomic profiling with SomaScan, and of these participants, 658 completed the 20-week exercise training program and had proteomic profiling performed after completion. We first examined the relationships between proteins that were significantly modified at both timepoints in the acute exercise cohort and cardiometabolic traits in HERITAGE, which included measures of body composition (BMI, waist-to-hip circumference ratio, visceral abdominal adiposity by computed tomography, and body fat percentage by underwater weighing), cardiorespiratory fitness (VO_2_max [ml O_2_/min/kg] and resting systolic blood pressure), lipids (high-density lipoprotein cholesterol, low-density lipoprotein cholesterol, and triglycerides), and glucose homeostasis (fasting glucose and insulin sensitivity [S_I_]). S_I_ was computationally estimated using the MINMOD software (https://www.cedars-sinai.edu/research/labs/bergman/resources.html) (44), which models the action of insulin on peripheral tissue using frequently sampled glucose and insulin levels obtained during an intravenous glucose tolerance test. We log_2_-transformed S_I_ prior to analysis. Aptamer abundance was log_2_-transformed and standardized to mean 0 and SD 1. We used linear mixed regression models with random effects of family grouping and fixed effects of age, sex, and race for body composition traits and age, sex, race, and BMI for the remaining traits. We then tested for change in these proteins before and after aerobic training in HERITAGE using a two-sided, one-sample *t* test. We corrected for multiple hypothesis testing using FDR <0.05 across 56 proteins tested (those that significantly changed at both timepoints in the acute exercise group). All analyses were performed in R 4.1.3 (R Foundation for Statistical Computing).

## Results

### Acute Exercise Cohort

The 75 acute exercise cohort participants were predominantly male (92%) and self-identified White (99%) with mean (SD) age 63 (9) years and BMI 29.4 (4.7) kg/m^2^ (Table 1). Most participants had CVD risk factors, such as hypertension (69%) and hyperlipidemia (59%), and some had a history of CVD (15%). On average, participants achieved an adequate level of exercise stress, as demonstrated by percent-predicted peak heart rate of 90% (14) and METs of 9.6 (2.6) *kcal/hr/kg*.

**Table:** Baseline characteristics of participants

Characteristic	Participants (N = 75)
Age	63 ± 9
Male	69 (92%)
White	74 (99%)
Body mass index, kg/m2	29.4 (26.0, 31.0)
Smoking status	
Current	4 (5%)
Former	35 (47%)
Never	36 (48%)
Hypertension	52 (69%)
Hyperlipidemia	59 (79%)
Diabetes	10 (13%)
Cardiovascular disease	11 (15%)
eGFR, ml/min/1.73 m2 (N = 57)	77 ± 17
Total cholesterol, mg/dl (N = 52)	168 (142, 204)
HDL-C, mg/dl (N = 52)	49 (42, 59)
Resting SBP, mmHg	128 (120, 139)
Resting DBP, mmHg	76 (70, 80)
Resting HR, beats/min	66 ± 11
Peak SBP, mmHg	172 (160, 189)
Peak DBP, mmHg	72 (68, 80)
Peak HR, beats/min	142 ± 24
Exercise duration, min	8.1 ± 2.6
Percent predicted peak HR, %	90 ± 14
Metabolic equivalent achieved, kcal/hr/kg	10.0 (7.5, 11.5)

Continuous variables are expressed as mean ± SD or median (IQR) for those with skewed distributions, and categorical variables are expressed as n (%). DBP, diastolic blood pressure; eGFR, estimated glomerular filtration rate; HDL-C, high-density lipoprotein cholesterol; HR, heart rate; SBP, systolic blood pressure.

### Protein Changes at Peak Exercise and 1-Hour Postexercise

We identified 765 unique proteins that significantly changed at peak exercise (“peak”) compared to baseline and 128 proteins that significantly changed at 1-h postexercise (“post”) compared to baseline. supplemental Table S1 shows the full results of changes at both timepoints for all aptamers tested as well as protein annotations (*e.g.*, Entrez Gene symbols, which will be used as abbreviations for all proteins) and supportive evidence of their aptamers’ specificity for the target proteins. Of the 863 aptamers that changed at either timepoint, 511 (59.2%) aptamers had *cis* pQTLs reported by at least one published study. In contrast, only 1516 (38.6%) of the 3926 aptamers that did not change at either timepoint had previously reported *cis* pQTLs. Among these 863 aptamers, 247 had correlations reported in comparative studies of the two platforms (29, 40, 41, 42), of which at least one study reported a correlation >0.8 for 45 (18.2%) aptamers and >0.6 for 125 (50.6%) aptamers. Overall, there was enrichment for protein-specific aptamer binding based on genetics and an orthogonal assay among the exercise-responsive proteins.

**Fig. 1:**
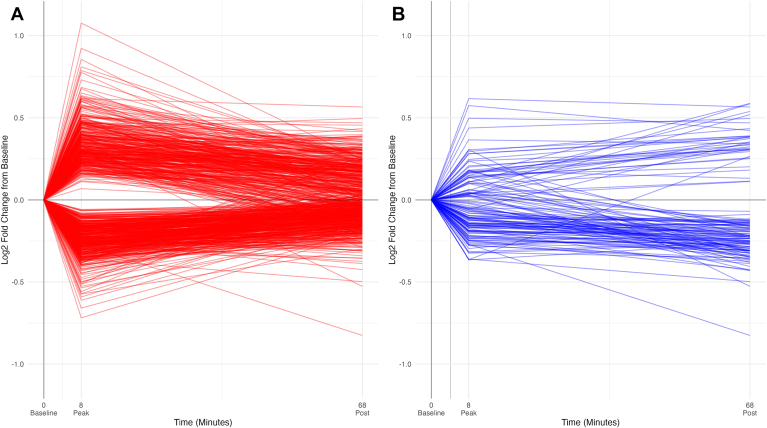
**Protein changes over time.***Panel A* shows a spaghetti plot in *red* of the mean log_2_ fold change for the 731 aptamers that changed significantly at peak exercise. *Panel B* shows a spaghetti plot in *blue* of the mean log_2_ fold change for the 132 aptamers that changed significantly at 1-h postexercise. Each line represents one aptamer.

**Fig. 2:**
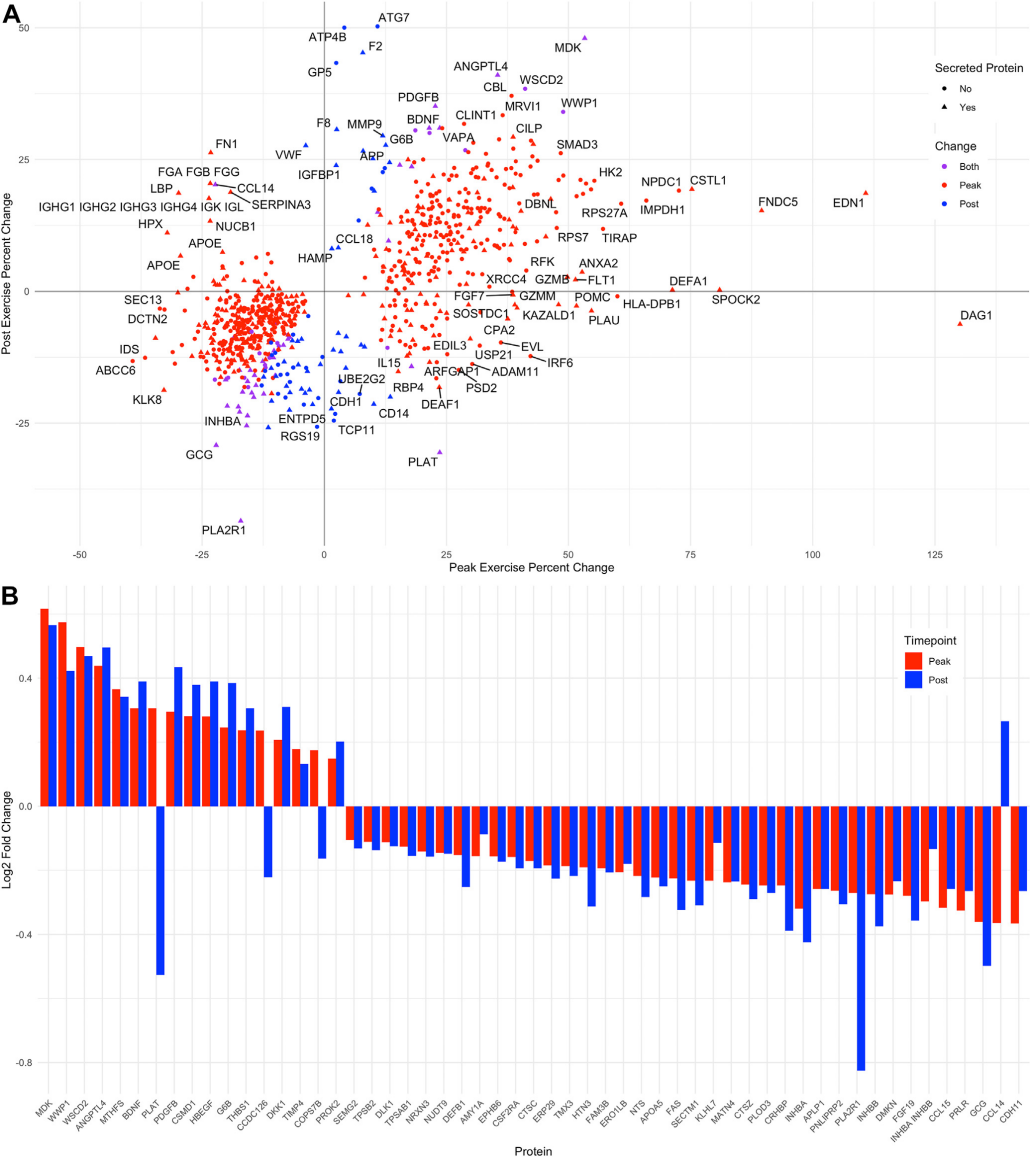
**Comparison of protein changes at peak exercise and postexercise.***Panel A* shows scatterplot of percent change from baseline of proteins that significantly changed at peak exercise (*red*), 1-h postexercise (*blue*), or both (*purple*). Proteins that are known to be secreted are marked with *triangles*, and the rest are marked by *circles*. *Panel B* shows a waterfall plot of log_2_ fold change from baseline for the 56 proteins that significantly change at both peak exercise (*red*) and 1-h postexercise (*blue*). Proteins are labeled with their Entrez Gene symbols.

At peak exercise, we observed that 354 proteins increased and 411 decreased, while 1-h postexercise, 34 proteins increased and 94 decreased relative to baseline. Figure 1 shows the trajectory over time of the proteins that changed at peak exercise in panel A (red) and proteins that changed postexercise in panel B (blue). The majority (92.7%) of proteins that significantly changed at peak exercise had nonsignificant changes from baseline to the 1-h postexercise timepoint, but nearly half (43.8%) of proteins that significantly changed 1-h postexercise also had significant changes at peak exercise. Figure 2*A* shows a scatterplot comparing all proteins that significantly changed either at peak exercise or postexercise and their absolute fold changes along each axis. Among the 56 proteins that changed at peak and postexercise, 52 (93%) had a concordant change at both timepoints (*i.e.*, increasing at peak and at postexercise or vice versa), and four proteins demonstrated discordant changes: tissue-type plasminogen activator (PLAT), coiled-coil domain-containing protein 126 (CCDC126), COP9 signalosome complex subunit 7b (COPS7B), and C–C motif chemokine 14 (CCL14) (Fig. 2*B*).

Based on their subcellular location as annotated by UniProt (45), the majority (62.5%) of proteins that changed postexercise were known secreted proteins compared to 34.5% of proteins that changed at peak exercise. In general, 35.5% of the SomaScan platform represents known secreted proteins. Therefore, while there are changes to many types of circulating proteins during peak exercise, proteins with more sustained changes were specifically enriched for compounds that may be actively secreted.

Supplemental Table S3 shows the relationship of proteins with clinical characteristics in the acute exercise cohort. Of the proteins that significantly change at either exercise timepoint, 113 proteins at peak exercise and nine proteins at 1-h postexercise had an association with METs achieved based on an unadjusted *p*-value <0.05 (supplemental Table S4). The proteins with the strongest associations at peak exercise include dystroglycan (DAG1), adhesion G protein–coupled receptor E2 (ADGRE2), and vascular endothelial growth factor receptor 1 (FLT1), all of which had higher abundance with higher METs achieved.

### Pathway Enrichment Analyses Among Acute Exercise Proteins

**Fig. 3:**
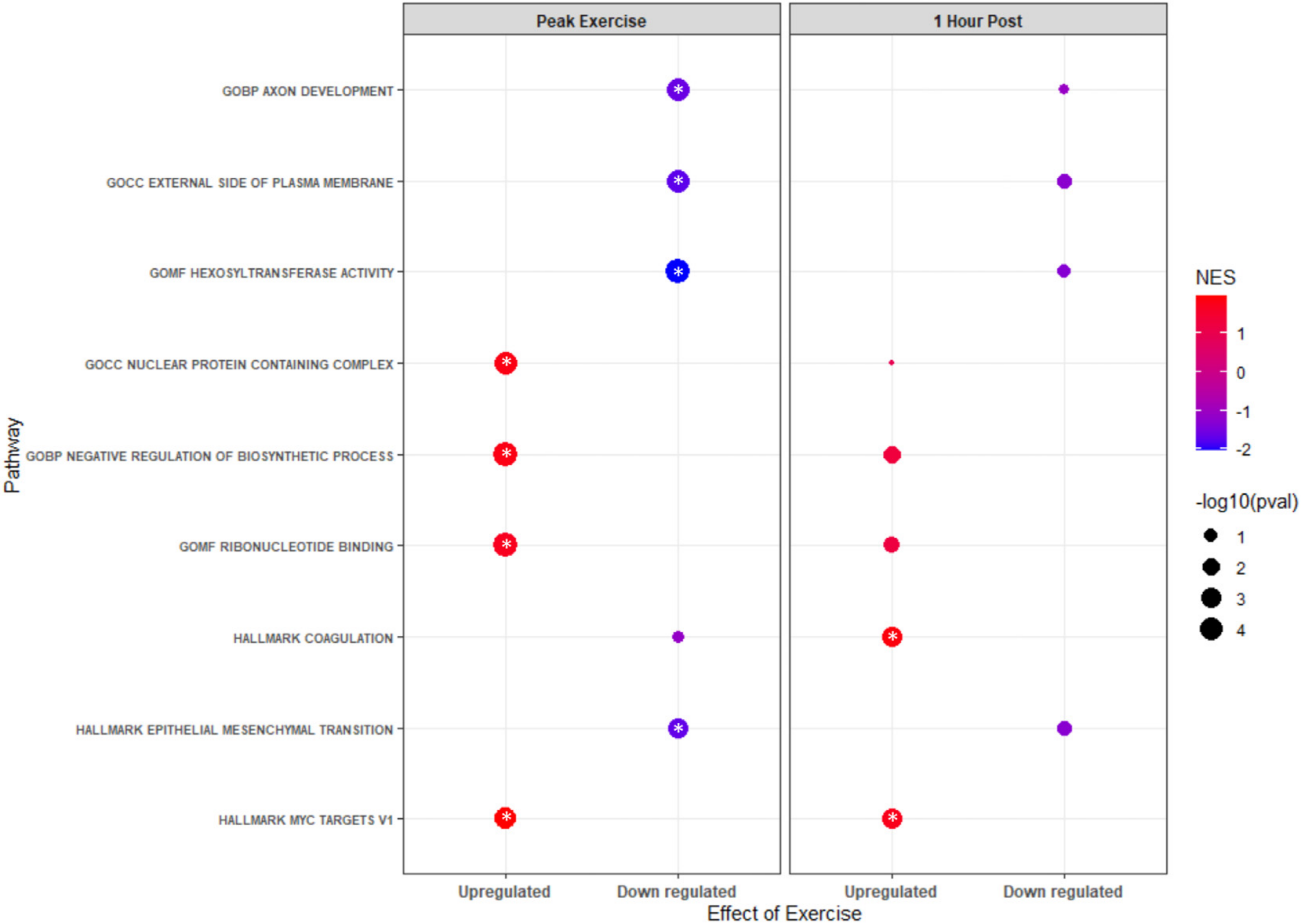
**Gene set enrichment analysis: Shown are hallmark or gene ontology pathways from gene set enrichment analysis that are significantly upregulated or downregulated at either peak exercise or 1 h postexercise.***Asterisks* inside the points indicate significance at false discovery rate <0.05. GOBP, gene ontology biological process; GOCC, gene ontology cellular component; GOMF, gene ontology molecular function.

Across all proteins measured, GSEA showed FDR significant enrichment and upregulation of the MYC targets’ hallmark pathway at both peak exercise and 1-h postexercise (Fig. 3). In addition, proteins involved in epithelial mesenchymal transition were significantly downregulated at peak exercise, and those related to coagulation were upregulated at 1-h postexercise. At peak exercise, 69 different GO sets were upregulated, and 14 sets were downregulated (supplemental Table S2). The most significantly upregulated GO sets for molecular function, biological process, and cellular component were ribonucleotide binding, negative regulation of biosynthetic process, and nuclear protein–containing complex, respectively. The most significantly downregulated GO sets for molecular function, biological process, and cellular component were hexosyltransferase activity, axon development, and external side of plasma membrane, respectively. No GO sets at 1-h postexercise showed significant enrichment.

### Protein-Cardiometabolic Traits Associations and Exercise Training Responses

We then further characterized proteins that had sustained changes at both acute exercise timepoints in HERITAGE, which measured a variety of cardiometabolic and body composition traits. At baseline, HERITAGE participants were younger (mean age 34 years), more diverse (55% female, 38% Black), and less overweight (mean BMI 26.5 kg/m^2^) compared to our acute exercise cohort (supplemental Table S5).

All 56 proteins that changed at both timepoints in the acute exercise cohort were measured in HERITAGE; 44 (79%) had significant associations with at least one cardiometabolic trait in HERITAGE at baseline, and 35 (63%) were associated with at least one measure of body composition or adiposity (supplemental Table S6). Additionally, the majority of proteins with sustained changes in the acute exercise cohort had supporting evidence for aptamer specificity, either in HERITAGE or in the literature. In a subsample of HERITAGE, 23 of the 56 proteins were also measured by Olink; 16 proteins (70%) had Spearman correlations >0.6 and 47 (84%) had at least one *cis* pQTL in the literature. Seven proteins (CCDC126, PLAT, amyloid-like protein 1 [APLP1], ephrin type-B receptor 6 [EPHB6], metalloproteinase inhibitor 4 [TIMP4], alpha-amylase 1 [AMY1A], and neurexin 3 beta [NRXN3]) had significant associations with six or more traits. CCDC126 was associated with nine different traits. Higher CCDC126 abundance was associated with lower BMI, waist-to-hip circumference ratio, visceral adiposity, body fat percentage, triglycerides, and glucose, and higher VO_2_max, high-density lipoprotein cholesterol, and S_I_.

**Fig. 4:**
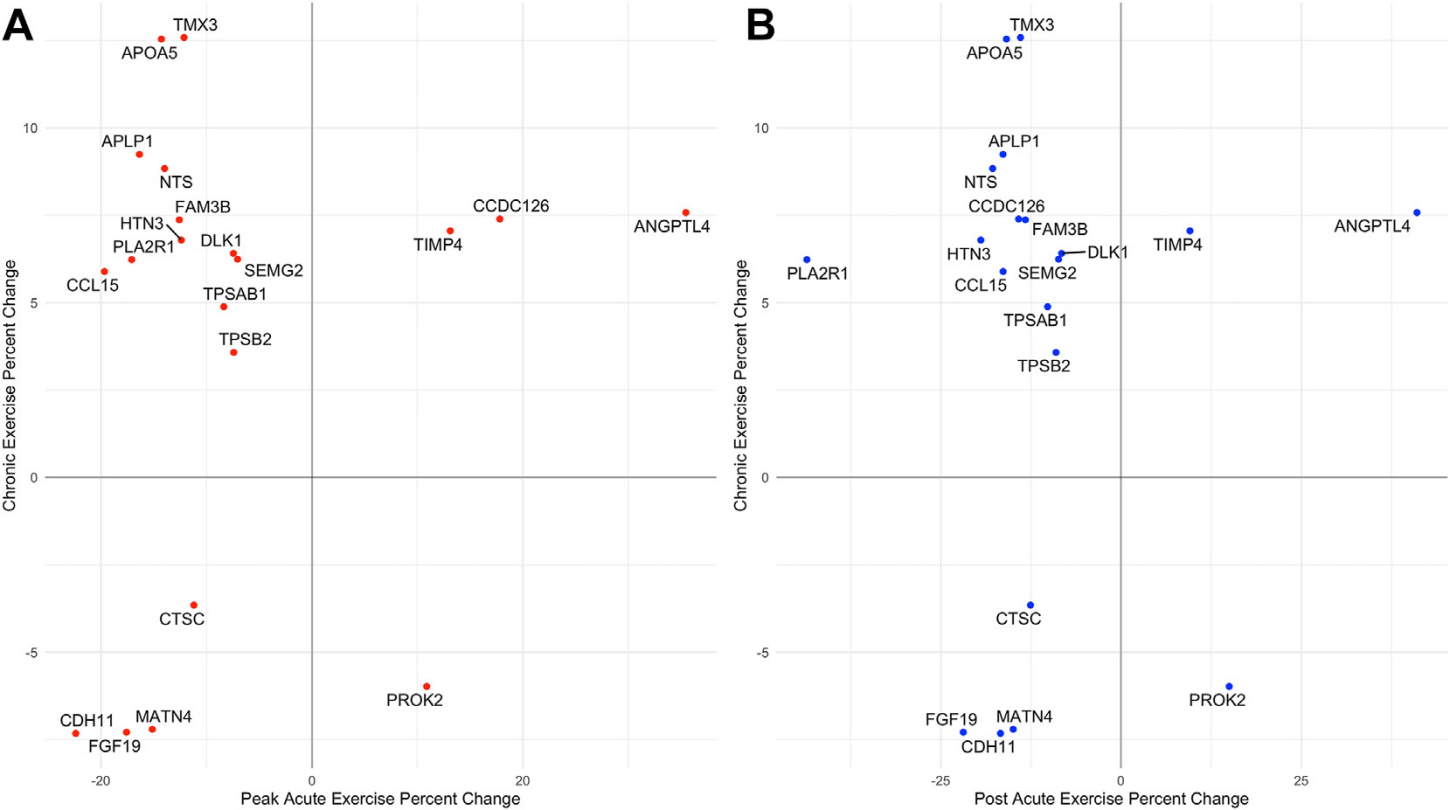
**Comparison of protein changes with acute exercise and chronic exercise.***Panel A* shows a scatterplot in *red* of percent change from baseline for proteins that significantly changed at peak exercise in the acute exercise cohort and with chronic exercise training in HERITAGE. *Panel B* shows a scatterplot in *blue* the percent change from baseline for proteins that significantly changed at postexercise in the acute exercise cohort and with chronic exercise training in HERITAGE. Proteins are labeled with their Entrez Gene symbols. HERITAGE, HEalth, RIsk factors, exercise Training, And Genetics.

Last, we examined whether proteins that have sustained response with a single exercise bout also change with chronic aerobic exercise. We found that 20 (36%) proteins showed a significant change after 20 weeks of training in HERITAGE (Fig. 4). In general, proteins that decreased with acute exercise increased with aerobic training. However, there were notable exceptions to this pattern, including angiopoietin-related protein 4 (ANGPTL4), TIMP4, fibroblast growth factor 19 (FGF19), matrilin 4 (MATN4), cadherin 11 (CDH11), and dipeptidyl peptidase 1 (CTSC), which either increased or decreased with both acute exercise and exercise training.

## Discussion

Acute exercise reflects a highly coordinated effort across multiple organ systems, including the skeletal muscle, neural, endocrine, and cardiopulmonary circuits (46). Previous studies have demonstrated significant plasma proteomic changes during acute exercise (21, 22, 23), highlighting the circulatory system’s function as a conduit for exercise-stimulated circulating biochemicals, some of which participate in mediating health benefits. Here, we expand upon previous discovery efforts by applying a more expansive plasma protein screen to identify 765 proteins that change at peak exercise. We further detail the proteomic responses in the postexercise recovery period and highlight 128 proteins that are changed 1-h postexercise and, in particular, 56 proteins with sustained changes (both peak and 1-h post). We show that proteins with sustained changes are enriched for being secreted and further characterize these proteins’ associations with cardiometabolic traits and changes with chronic exercise in a chronic exercise cohort.

### Pathway Changes During Peak Exercise

Our acute exercise findings recapitulate those previously made by Guseh *et al*. who examined 1305 proteins (measured by an older SomaScan platform) in 12 physically fit young men immediately following moderate- or high-intensity endurance exercise (21). We confirmed 148 of their peak exercise protein findings, including those with known relations to exercise such as brain-derived neurotrophic factor and ANGTPL4 (47, 48). Additional overlapping proteins include multiple WNT signaling modulators (dickkopf-1, 3, and 4 [DKK1, 3, 4]), various interleukin proteins and their receptors, and apolipoprotein A1. Despite differences in exercise duration (mean 8 min *versus* 30–50 min), exercise intensity, and study population (mean age 63 *versus* 21), the overlapping proteins strengthen the evidence for these proteins being “exercise responsive.” Furthermore, we found similar direction of change at peak exercise for 68% of the 1268 overlapping proteins examined by us and Guseh *et al*. (21).

More importantly, we identified hundreds of proteins altered during peak exercise that have not previously been reported including DAG1, testican 2 (SPOCK2), kallikrein 8 (KLK8), FLT1, and signal transducer and activator of transcription 1-alpha/beta. DAG1—the most abundantly increased protein at peak exercise—is a central component of the dystrophin–glycoprotein complex that links the extracellular matrix and the cytoskeleton in skeletal muscle (49), and its alpha subunit is an extracellular receptor for agrin and promotes the aggregation of acetylcholine receptors (50). Despite its greater than 2-fold change at peak exercise, DAG1 returned to baseline with a nonsignificant 6% decrease 1-h postexercise. Similarly, most proteins (709/765, 93%) that had significant peak exercise alterations had resolved changes at 1-h postexercise, and 65% of those proteins had no known extracellular function. These observations suggest that there may be flow-mediated or nonspecific perturbations to the circulating proteome that occurs at peak exercise, which need to be cautiously interpreted and studied further on an individual protein level. It is important to note that our study recruited mostly untrained individuals to exercise on a treadmill, and running without habitual running experience may cause more muscle damage and stress response than after repeated bouts. While we also identified many proteins whose peak abundance change related to METs achieved, it is difficult to know how much change was a function of exercise intensity *versus* the individual’s intrinsic cardiorespiratory fitness.

### Proteomic Alterations in the Postexercise Recovery Period

We observed 128 proteins that significantly changed 1-h postexercise relative to baseline. Most proteins that changed postexercise also changed at peak exercise in a concordant direction, the most significantly decreased protein being secretory phospholipase A2 receptor (PLA2R1), a transmembrane glycoprotein involved in cell proliferation and migration (51). We also report proteins such as fibrinogen-like protein 1, a liver-secreted protein implicated in protection against exogenous injury (52), which did not change at peak exercise but decreased 1-h post. Exercise has previously been shown to increase renal clearance of proteins by as much as 40-fold (53), which may explain observed decreases in plasma proteins postexercise. However, of the 94 proteins decreased 1-h postexercise, only 10 were associated with estimated glomerular filtration rate (calculated from age, sex, and creatinine (54)) at baseline (*p* < 0.05, supplemental Table S3), suggesting that most protein changes are not entirely the byproduct of increased renal clearance. Alternative mechanisms could include active uptake of proteins during tissue repair or suppressed gene expression. Of the proteins with significant postexercise changes, the majority have not been described, and two—von Willebrand factor and heart-type fatty acid–binding protein—were previously reported at 1-h postexercise with concordant direction of change (23).

Across all proteins, GSEA identified a significant enrichment for upregulation of coagulation-related proteins postexercise. Exercise has been shown to increase procoagulant activity (55, 56), but this increase in coagulation is typically thought to be balanced by an increase in fibrinolytic activity. Here, we show a robust upregulation of proteins involved in coagulation 1-h postexercise with no concomitant increase in fibrinolysis. Circulating procoagulant proteins such as thrombin, coagulation factor VIII, matrix metalloproteinase-9, thrombospondin-1, and von Willebrand factor were increased 1-h postexercise, while PLAT was significantly decreased. Additionally, there was no effect on coagulation during peak exercise, which suggests that activation of the coagulation pathways may be more specific to the postexercise recovery period. These findings require further confirmation.

### Sustained Proteomic Changes After Exercise Were Associated With Cardiometabolic Traits

At 1-h postexercise, most alterations in the proteome seen at peak exercise returned to basal levels, but 56 proteins’ abundance remained perturbed. Among the proteins with sustained changes, we observed an enrichment for secreted proteins, which supports their role as biologically relevant circulating factors. The 56 proteins that were significantly changed at both timepoints include known exerkines highlighted above, brain-derived neurotrophic factor, and ANGPTL4, as well as several proteins with unknown roles in exercise. Almost all proteins with sustained changes following exercise in the current study were associated with cardiometabolic traits in HERITAGE, and several were also significantly altered by exercise training.

Across all proteins, GSEA showed significant enrichment and upregulation at both timepoints for MYC targets, a network of proteins that may be responsive to altered metabolic demands (57) and exerts fine-tuning effects on glycolysis, protein synthesis, and gene transcription (58, 59). The MYC network has been hypothesized to influence wide-spread gene expression during exercise through a modulatory effect of lactate (59), a known exerkine (10). We were unable to evaluate the correlation between the increase in circulating MYC network proteins and lactate during exercise in this study, and further work is needed to elucidate how the MYC network may mediate biological responses to exercise.

### Proteomic Changes With Acute and Chronic Exercise

Proteins that decreased at both timepoints and increased with chronic exercise included PLA2R1, histatin 3 (HTN3), and APLP1. All three proteins were inversely associated with measures of body composition and adiposity in HERITAGE. PLA2R1 has been suggested to promote brown adipocyte fate and improve mitochondrial function (60). While APLP1 has traditionally been studied in the context of Alzheimer’s disease (61), obesity in mice is associated with decreased hippocampal expression of APLP1 (62), which is consistent with our observations of its adiposity associations in HERITAGE. In addition, APLP1 was inversely related to low-density lipoprotein cholesterol, triglycerides, and glucose even after adjusting for BMI, and despite decreasing with acute exercise, it was significantly increased with chronic training. Interestingly, proteins that changed with acute exercise and responded to chronic exercise often displayed discordant responses, with those proteins decreased by acute exercise increased by exercise training or vice versa. The significance of this general trend is unclear, but it could reflect relationships akin to exercise’s impact on inflammation and interleukin-6, which is released during exercise but decreased with training (11). It is important to note that differences in the two cohorts make precise comparisons challenging, and further studies of acute exercise and chronic training in the same individuals are needed (and in progress) (16).

In contrast to many of the proteins measured, FGF19 and CCDC126 changed in a concordant pattern during acute and chronic exercise. FGF19 has been implicated as an endocrine protein regulating energy balance and glucose metabolism that may be decreased in circulation following resistance exercise (63). Results from the current study support an exercise-induced decrease in FGF19 during acute aerobic exercise and in response to endurance training. However, FGF19 was inversely associated with all measures of adiposity in HERITAGE. One possible explanation for these seemingly incongruent findings is that if FGF19 is causally related to metabolic health, and exercise’s favorable metabolic adaptations suppress the feedback for its upregulation, suggesting that it is a marker of exercise’s biological effects and not a mediator—a hypothesis that needs further testing. Despite CCDC126 having associations with cardiometabolic traits across all domains tested—body composition, cardiorespiratory fitness, lipids, and glucose homeostasis, very little is known about its physiological function. In one analysis, CCDC126 had strong genetic colocalization with monocyte count (36), suggesting a relationship with immune function, and other proteins related to the immune system have been found to change with acute exercise as well (22). Its multiple positive cardiometabolic associations and increase with chronic exercise position it as a promising candidate marker for healthy metabolic changes.

### Limitations and Future Directions

The current study has several limitations. First, the lack of a control group limits our ability to conclude definitively that observed proteomic changes resulted from exercise alone. Contrepois *et al*. recently demonstrated that most circulating analytes, including proteins, do not change within a 1-h time period in control participants (23), supporting the consistency of most of our findings. Furthermore, the choice of timepoints was constrained by participant convenience and willingness, and more frequent plasma sampling at different time intervals are needed to identify biologically relevant kinetics. Second, our acute exercise clinical sample consisted largely of middle-aged White males with some CVD risk factors, and it may not be representative of broader populations, as previous investigations have shown differential protein responses to exercise in the context of type 2 diabetes (64). Our findings may also be unique to a clinical exercise protocol that is designed to evaluate coronary heart disease. Within our cohort, there was a wide range of exercise durations on the protocol and peak METs achieved, which decreases our ability to detect proteomic changes that require specific exertion thresholds. Furthermore, we could not identify the tissue sources of circulating proteins in this experiment. Ongoing efforts through the Genotype-Tissue Expression project and the molecular transducers of physical activity consortium (16) hold promise in this regard. Molecular transducers of physical activity consortium will also address an additional limitation of our study, in which we were unable to confirm whether proteins that change with acute and chronic exercise would be observed within the same individual. One focus of future investigation will be to reconcile proteins in pathways that appear to have discordant direction of change in the acute and chronic settings. Last, while we provide supplemental data to support SomaScan aptamers’ specificity as protein probes, many aptamers likely poorly reflect true protein abundance, and gold standard assays are needed to corroborate our findings.

## Conclusion

By profiling three timepoints—baseline, peak exercise, and 1-h postexercise—with a large proteomics platform, our study represents an early step toward the biochemical characterization of acute exercise protein kinetics in the blood. Additionally, we provide complementary evidence linking perturbed proteins to cardiometabolic phenotypes and chronic exercise response and offer one way to prioritize exercise-responsive proteins for further investigation based on their potential relevance to cardiometabolic health and relation with chronic exercise.

## Data Availability

Data supporting the findings of this study are available from the corresponding author upon reasonable request.

## Supplemental data

This article contains supplemental data (31, 32, 33, 34, 35, 36, 37, 38, 40, 41, 42).

## Conflict of interest

The authors declare no competing interests.
