# Supplementary material for: Plasma Proteomic Kinetics in Response to Acute Exercise
Source: Mol Cell Proteomics. 2023 Jun 19;22(8):100601. doi: 10.1016/j.mcpro.2023.100601 (PMC10460691; doi:10.1016/j.mcpro.2023.100601)
Supplement: Supplemental Figure 1 [file mmc1.pdf]

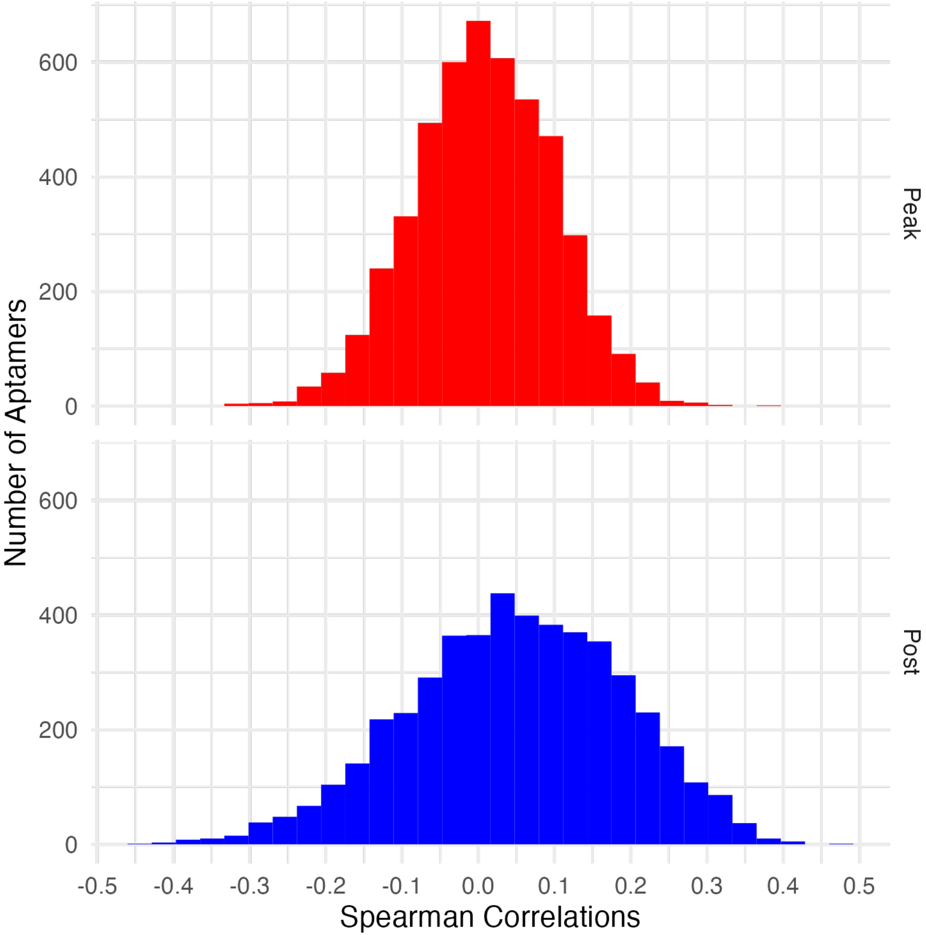

Supplemental Figure 1: Figure shows the distribution of Spearman correlations for each aptamer between aptamer fold change and the duration of storage (days) at peak and 1-hour post-exercise ("post").
